# Impact of different neurectomy techniques on managing chronic pain after inguinal hernia repair: a meta-analysis and systematic review

**DOI:** 10.1007/s10029-025-03438-0

**Published:** 2025-08-12

**Authors:** Emmanouil Charitakis, Eyman Haj-Ali, Farah Al Hasani-Pfister, Baraa Saad, Niklas Ortlieb, Amanda Haberstroh, Florian Ponholzer, Stephanie Taha-Mehlitz, Lisa-Marie Schupp, Robert Christian Bauer, Sebastian Lamm, Daniel M. Frey, Robert Rosenberg, Anas Taha

**Affiliations:** 1 Faculty of Medicine, University of Basel, Basel, Switzerland; 2 Emergency Department, Al-Habib Medical Group, Riyadh, Saudi Arabia; 3 Department of Visceral Surgery, Cantonal Hospital, Baselland, Switzerland; 4 Faculty of Medicine, Royal Lancaster Infirmary, Lancaster, UK; 5 Medoc Swiss, Data Science and Statistics, Basel, Switzerland; 6 Laupus Health Sciences Library, East Carolina University, Greenville, NC USA; 7 Department of Visceral, Transplant, and Thoracic Surgery, Center of Operative Medicine, Medical University of Innsbruck, Innsbruck, Austria; 8 Clarunis, University Centre for Gastrointestinal and Liver Diseases, St. Clara Hospital and University Hospital, Basel, 4002 Switzerland; 9 Department of Visceral Surgery, Cantonal Hospital Baden, Baden, Switzerland; 10 Department of Surgery, Brody School of Medicine, East Carolina University, Greenville, NC USA; 11 Department of Biomedical Engineering, Faculty of Medicine, University of Basel, Allschwil, 4123 Switzerland

**Keywords:** Chronic pain, Postherniorrhaphy, Neurectomy, Inguinal hernia, Pain management, Meta-analysis

## Abstract

**Purpose:**

This meta-analysis aims to analyze the outcomes of neurectomy for treating chronic post herniorrhaphy pain (CPIP), with a focus on the efficacy of various surgical approaches (open, laparoscopic transabdominal, endoscopic retroperitoneal and combined approach), and types of neurectomy—single, double and triple.

**Methods:**

The research was registered with PROSPERO (CRD42023475401). A search in MEDLINE, Embase, Scopus, and Cochrane Central databases was conducted from the inception to November 2023. All participants aged 18 years and older who underwent neurectomy for treating CPIP were included. PRISMA guidelines were followed, selecting only randomized controlled trials, cohort studies and case series. A random-effects model was used for single-arm analyses, while the inverse variance and Mantel-Haenszel methods were employed for two-arm analyses.

**Results:**

The analysis includes fifteen studies involving 701 patients. Following neurectomy, 90% of the patients experienced an improvement in pain overall. Only 9.4% of the patients had postoperative complications. Among the surgical types, triple neurectomy demonstrated the highest overall pain improvement rate at 98.2%. Double neurectomy showed the highest rate of complete pain relief at 80.1%, but it also had a higher complication rate of 15.3%. In terms of surgical approach, the endoscopic retroperitoneal method not only had the highest overall improvement rate of 95.5% compared to other approaches but also the highest complication rate of 28.7%.

**Conclusion:**

Neurectomy was shown to be an effective treatment for neuropathic CPIP across various surgical techniques. Randomized controlled trials would be of vital importance to facilitate the evolution of surgical strategy and patient outcomes.

**Supplementary information:**

The online version contains supplementary material available at 10.1007/s10029-025-03438-0.

## Introduction

Inguinal hernia repair is a commonly carried out surgical procedure. The most frequent complication of this surgery, hernia recurrence, has been significantly reduced by advances in surgical techniques, particularly the tendency to utilize mesh repair approaches. Despite this, persistent groin discomfort following hernia surgery continues to be a major concern with its impact on patients’ quality of life [1–5].

Chronic post herniorrhaphy pain (CPIP) can be both nociceptive and neuropathic pain. Nociceptive pain arises from tissue damage or inflammatory responses, while neuropathic pain is due to nerve injury, entrapment or compression by a foreign body or scar tissue, leading to persistent pain even in the absence of a noxious stimulus. The complexity of this pain syndrome arises due to multiple nerve interactions at the inguinal region, where the ilioinguinal, iliohypogastric, and genitofemoral nerves are vulnerable [2, 3]. The reported incidence of chronic pain varies widely in the literature, ranging from 0.7 to 43.3% [6]. This variation is largely due to differences in definitions of chronic pain, the timing of assessments, and the measurement methods used [6]. The prevalence of debilitating pain, which interferes with daily activities or work, is between 0.5% and 6% [7]. In addition to lowering the patient’s quality of life, chronic pain also has social and financial effects on healthcare systems [1, 3]. Typically, if there has been no improvement in CPIP after several months, a multidisciplinary approach incorporating pharmacological, behavioural, and interventional methods, including nerve blocks is employed. If conservative treatments fail, surgical management can be indicated [8].

In 1982, Stulz and Pfeiffer first described surgical management as a safe and effective therapy for postoperative groin discomfort [9]. Since then, several studies have established the effectiveness of surgical therapy for nerve damage that occurred during or following lower abdominal or inguinal surgery. With differing success rates across studies, triple neurectomy of the ilioinguinal, iliohypogastric, and genitofemoral nerves has been proposed as a possible treatment for persistent neuropathic pain [1, 3, 10]. However, other studies suggest that double neurectomy may be better than triple neurectomy with fewer complications [11–13]. It is still unclear which of these neurectomy types should be used. Furthermore, there is still uncertainty on the best surgical approach—open, laparoscopic transabdominal, endoscopic retroperitoneal, or combination of both approaches [14–16].

This meta-analysis aims to examine the outcomes of neurectomy surgery for CPIP, with a focus on the efficacy of different neurectomy types and surgical approaches.

## Methods

### Search strategy and data sources

A comprehensive search was performed across MEDLINE (PubMed), Embase (Elsevier), Scopus (Elsevier), and Cochrane Central databases from their inception through November 2023. The search strategy was developed and executed by a health sciences librarian (AH) in collaboration with a reviewer (BS), utilizing both keywords and controlled vocabulary related to terms such as “denervation,” “neurectomy,” “nerve resection,” “postherniorrhaphy,” “hernioplasty,” “neuralgia,” “inguinodynia,” “neuropathy,” “orchialgia,” “pain,” “laparoscopy,” “endoscopy,” and “retroperitoneal.” This review was prospectively registered with PROSPERO (CRD42023475401). Database search results were imported into Covidence review software for the deduplication process. Following Cochrane Systematic Review Guidelines, two reviewers (EHA and EC) independently screened 439 titles and abstracts, as well as 56 full texts, against the predefined eligibility criteria. Disagreements were resolved by a third independent reviewer (BS).

### Eligibility criteria and quality assessment

Eligible studies must have met all the following inclusion criteria: (1) individuals over 18 years old experiencing chronic inguinal pain post-hernia repair surgery, (2) individuals who underwent neurectomy surgery for treating CPIP (3) studies reporting primary outcomes related to neurectomy surgery. Randomized control trials, prospective cohort, retrospective cohort and case series studies were included. Case reports, abstracts and poster presentations were excluded. Inclusion was restricted to only English-language articles. The methodological quality of each study was independently evaluated by two authors (EHA and EC) using the ROBINS-I tool for non-randomized studies and the ROB2 tool for randomized studies [17, 18]. Disagreements were resolved by a third independent reviewer (BS).

### Statistical analysis

In single-arm analyses, a random-effects model and a generic inverse variance method of DerSimonian and Laird were employed to pool means of continuous variables and rates of binary outcomes [19]. Proportions were transformed using the logit transformation before the meta-analysis was conducted. For two-arm analyses, means and proportions were pooled using the inverse variance method for continuous data and the Mantel-Haenszel method for dichotomous data [20]. The weight of each study was assigned based on its variance. The heterogeneity of effect size estimates across studies was quantified using the I² index, with significance set at (*p* < 0.05). The I² values were interpreted as indicating minimal heterogeneity (0–25%), moderate heterogeneity (26–50%), or substantial heterogeneity (51–100%) [21]. Data analysis was performed using Open Meta Analyst software (CEBM, Brown University, Providence, Rhode Island, USA) for single-arm analyses and two-arm analyses. If mean or standard deviation (SD) was unavailable, the median was converted to mean and the range, interquartile range or confidence intervals were converted to SD using the formulas from the Cochrane Handbook for Systematic Reviews of Interventions [20].

### Endpoints

Pain was defined both by duration and type, with chronic pain characterized variably across studies. Chronic postoperative pain was defined as lasting more than six months in three studies [1, 3, 16], and more than three months in six studies [2, 5, 11, 13, 14, 22]. Neuropathic pain or neuralgia diagnosis was primarily based on clinical features, including pain in the inguinal region radiating to areas like the hemiscrotum or labium majus, or pain aggravated by activities such as walking or stooping and relieved by rest or thigh flexion, or pain accompanied by paresthesia, hypoesthesia, and dysesthesia [10, 12, 23, 24]. Diagnostic methods used included Tinel sign, dermatome sensory testing, response to anesthetic blocks as well as preoperative diagnostic imaging (MRI, CT, or ultrasound) to exclude other conditions and identify potential mesh-related complications [1–3, 11, 13, 16, 22, 24, 25].

Surgical approaches varied. Open neurectomy involves a single incision for direct access to the abdominal cavity and often involved extending the old incision and performing nerve resection [2, 5, 10, 11, 13, 23, 25]. The laparoscopic transabdominal approach involved laparoscopic access to the abdominal cavity through transabdominal ports to access and resect the nerves [14, 16]. The endoscopic retroperitoneal approach involves laparoscopic entry into the retroperitoneal space without direct access to the abdominal cavity. The patient is commonly in the lateral decubitus position allowing for the definition of the lumbar plexus and proximal nerve resection over the quadratus lumborum and psoas muscles [1, 3, 12]. The combined approach integrates laparoscopic exploration and open surgery allowing surgeons to tailor the intervention based on intraoperative findings, preoperative findings and previous surgeries [4, 22, 24].

Resected nerves included the ilioinguinal (IIN), iliohypogastric (IHN), genitofemoral (GFN), and lateral femoral cutaneous (LFCN), with neurectomy performed in single, double, or triple forms, where single targets one nerve, double targets two (typically IIN and IHN), and triple, involves resection of IIN, IHN, and GFN.

Improvement in pain was our primary outcome measured using self-reporting methods and scales. Pain improvement was classified into two main categories: pain improved, and pain not improved. To allow for a more detailed analysis, pain improvement was further stratified into four subcategories: Free of Pain, which denotes complete resolution of pain; Partial Improvement, where patients experienced meaningful reductions in pain, but did not achieve full relief; Pain the Same, where patients reported no change in pain levels; and Pain Worsened, where patients experienced an increase in pain following the procedure. Where possible, pain was assessed using the Visual Analog Scale (VAS), where a score of 0 represents no pain and a score of 10 indicates the worst pain [26]. The secondary endpoint of the study was complications.

##  Results

### Study selection

The initial search yielded 773 database records. Which were initially sorted by Covidence, which automatically identified and removed 334 duplicates. Then two reviewers (EHA and EC) independently screened 439 titles and abstracts, as well as 56 full texts, against the predefined eligibility criteria. From which 15 unique studies involving 701 patients met the eligibility criteria [1–5, 10–14, 16, 22–25]. Details of the study selection process and the PRISMA flow diagram are depicted in Supplementary Fig. 1.

### Risk of bias

Results of the quality assessment of all included studies are shown in Supplementary Fig. 2. In ROBINS-1, 6 studies were judged to have a moderate risk of bias [4, 11, 13, 14, 16, 24], while 8 studies were judged to have a serious risk of bias [1–3, 10, 12, 22, 23, 25]. In ROB2, one study was judged to have a high overall risk of bias [5].

### Baseline and procedural characteristics

The baseline characteristics of the included patients and studies are comprehensively described in Table 1. A total of 701 patients underwent 704 neurectomy surgeries. The pooled proportion of male patients was 86.6% (*n* = 630; 95% CI: 79.3, 91.6; I^2^ = 51.6%) [1, 3–5, 10–14, 23–25]. The mean age of participants was 45.7 years (*n* = 219; 95% CI: 42.4, 49.0; I^2^ = 83.6% [1, 3–5, 11, 13, 14, 16, 22, 24, 25]. The pooled proportion of patients who underwent primary hernia surgery laparoscopically was 16.9% (*n* = 640; 95% CI: 6.20, 38.3; I^2^ = 89.1%) [1–5, 11, 14, 23–25], and the pooled proportion who had open primary hernia surgery was 81.5% (*n* = 640; 95% CI: 58.7, 93.1; I^2^ = 90.2%) [1–5, 11, 14, 23–25]. The pooled proportion of patients with unilateral groin pain was 95.1% (*n* = 228; 95% CI: 90.5, 97.6; I^2^ = 0%) [1, 2, 5, 11–14, 16, 24, 25]and the pooled proportion with bilateral groin pain was 5.4% (*n* = 185; 95% CI: 2.6, 10.7; I^2^ = 0%) [1, 2, 5, 12–14, 16, 24, 25]. The pooled proportion of patients with right-sided inguinal pain was 66.0% (*n* = 60; 95% CI: 50.2, 78.8; I^2^ = 19.6%) [5, 13, 14, 16], and with left-sided inguinal pain was 34.0% (*n* = 60; 95% CI: 21.2, 49.8; I^2^ = 19.6%) [5, 13, 14, 16]. The pooled mean duration of pain post hernia surgery before neurectomy was 20.73 months (*n* = 163; 95% CI: 13.5, 27.9; I^2^ = 83.4%) [1, 5, 11, 13, 24, 25]. The pooled proportion who underwent prior failed operative non-neurectomy interventions was 20.7% (*n* = 144; 95% CI: 7.9, 44.3; I^2^ = 72.1%) [1–3, 14, 16, 25], while the pooled proportion of patients who underwent previously failed neurectomy was 10.1% (*n* = 160; 95% CI: 5.80, 16.8; I^2^ = 0%) [1–3, 10, 14, 16, 25].

**Table 1:** Baseline characteristic of included studies and patients

Study	No. Patients	No. of Procedures	Gender (Male)	Mean Age (Years) ± SD	Type of Hernia Repair Surgery		Bi/Unilateral Groin Pain		Side of Inguinal Pain		Mean Duration of Pain Post Hernia Surgery (Months) ± SD	Total Number of Operative Treatment	
					Laparoscopic	Open	Bilateral	Unilateral	Right	Left		Prior Failed Neurectomy Interventions for Inguinal Pain	Prior Failed Operative Interventions for Inguinal Pain
Amid 2007	415	415	383	NR ^a^	8	407	NR	NR	NR	NR	NR	NR	NR
Amid 2011	16	16	16	NR	NR	NR	NR	NR	NR	NR	NR	1	NR
Bjurström 2017	10	10	9	46.5 ± 9.8	6	4	0	10	NR	NR	31.2 ± 18.6	1	3
Campanelli 2013	46	46	NR	NR	0	46	0	46	NR	NR	NR	0	0
Chen 2013	20	20	14	46 ± 11.8	10	10	NR	NR	NR	NR	NR	4	12
Ducic 2008	18	18	14	45.6 ± 10.8	NR	NR	0	18	9	9	43.4 ± 33.8	NR	NR
GutiérrezCarrillo 2023	7	7	NR	48 ± 3.3	NR	NR	1	6	5	2	NR	0	0
Karampinis 2017	7	8	5	57.1 ± 11.8	2	6	1	6	7	1	NR	0	0
Keller 2008	18	18	NR	41 ± 7.3	NR	NR	NR	NR	NR	NR	NR	NR	NR
Loos 2010	54	56	43	50 ± 17.5	10	36	2	52	NR	NR	30 ± 74.3	5	14
Muto 2005	5	5	5	NR	NR	NR	0	5	NR	NR	NR	NR	NR
Rosen 2006	11	11	10	41 ± 5.5	0	11	1	10	NR	NR	14.3 ± 8.5	NR	NR
Valvekens 2015	4	4	4	43 ± 10.4	3	1	NR	NR	NR	NR	NR	NR	NR
Verhagen 2018	27	27	27	53 ± 12.5	0	27	0	27	19	8	12 ± 29.8	NR	NR
Vuilleumier 2009	43	43	39	35 ± 14.5	12	31	NR	43	NR	NR	12 ± 1.3	NR	NR

^a^ NR = Not Reported

Procedural characteristics of neurectomy surgeries are shown in Table 2. The total mean operative time was 92.1 min (*n* = 96; 95% CI: 71.5, 112.7; I^2^ = 98.3%) [1, 3, 11, 12, 16, 24], and the mean hospital postoperative stay was 1.00 days (*n* = 82; 95% CI: 1.00, 1.00; I^2^ = 67.4%) [3, 11, 12, 14, 16]. In the pooled proportions of types of neurectomy surgery, 6.8% of procedures were single neurectomy surgeries (*n* = 673; 95% CI: 2.00, 20.7; I^2^ = 85.9%) [1–3, 10–14, 16, 22–25], 26.6% were double neurectomy (*n* = 673; 95% CI: 10.9, 51.8; I^2^ = 83.5%) [1–3, 10–14, 16, 22–25], and 62.0% were triple neurectomy procedures (*n* = 673; 95% CI: 29.5, 86.4; I^2^ = 88.7%) [1–3, 10–14, 16, 22–25]. In the pooled proportion of surgical approaches, 64.7% were open surgeries (*n* = 704; 95% CI: 11.3, 85.9; I^2^ = 84.26%) [1–5, 10–14, 16, 22–25], 4.90% were laparoscopic transabdominal surgeries (*n* = 704; 95% CI: 1.40, 15.9; I^2^ = 67.9%) [1–5, 10–14, 16, 22–25], 7.30% were combined open and laparoscopic surgeries (*n* = 704; 95% CI: 1.70, 26.0; I^2^ = 77.2%) [1–5, 10–14, 16, 22–25], and 7.80% were endoscopic retroperitoneal neurectomy procedures (*n* = 704; 95% CI: 1.80, 28.4; I^2^ = 77.8%) [1–5, 10–14, 16, 22–25]. The pooled proportion of patients with IIN resection was 91.4% (*n* = 242; 95% CI: 78.5, 96.8; I^2^ = 66.7%) [1, 2, 5, 11–14, 22, 24, 25]. The pooled proportion of patients with IHN resection was 60.0% (*n* = 242; 95% CI: 26.8, 86.0; I^2^ = 89.0%) [1, 2, 5, 11–14, 22, 24, 25]. GFN resection occurred in 56.8% of patients (*n* = 242; 95% CI: 32.4, 78.3; I^2^ = 82.5%) [1, 2, 5, 11–14, 22, 24, 25]. LFCN resection occurred in 7.20% of patients (*n* = 186; 95% CI: 1.90, 24.2; I^2^ = 65.7%) [1, 2, 5, 11–14, 22, 24].

**Table 2:** Procedural characteristics of neurectomy surgeries

Study	Mean Operation Time (Minutes) ± SD	Mean Hospital Stay (Days) ± SD	Type of Neurectomy Surgery				Approach of Neurectomy Surgery				Neurectomy Branch						
			No. Patients	Single	Double	Triple	Open	Transb- dominal	Combined	Retroperi-toneal	No. Resected Nerves	INN	IHN	GFN	GFN Genit-al b.	GFN Femor-al b.	LFCN
Amid 2007	NR ^a^	NR	415	0	0	415	415	0	0	0	NR	NR	NR	NR	NR	NR	NR
Amid 2011	NR	NR	16	0	2	14	16	0	0	0	NR	NR	NR	NR	NR	NR	NR
Bjurström 2017	106.2 ± 18.3	NR	10	0	0	10	0	0	0	10	30	10	10	10	NR	NR	0
Campanelli 2013	NR	NR	46	2	0	44	46	0	0	0	134	46	44	44	NR	NR	0
Chen 2013	132.6 ± 27.2	0.8 ± 0.9	20	0	0	20	0	0	0	20	64	NR	NR	NR	NR	NR	NR
Ducic 2008	NR	NR	18	4	6	8	18	0	0	0	40	18	6	12	12	0	4
GutiérrezCarrillo 2023	101.4 ± 13.5	1 ± 0	7	0	0	7	0	7	0	0	NR	NR	NR	NR	NR	NR	NR
Karampinis 2017	NR	3.3 ± 2.5	7	0	6	2	0	8	0	0	18	2	0	8	0	1	8
Keller 2008	NR	NR	18	2	9	7	0	0	18	0	41	18	16	7	7	0	0
Loos 2010	NR	NR	54	39	12	5	56	0	0	0	78	44	9	25	NR	NR	NR
Muto 2005	55 ± 3.8	1 ± 0	5	0	5	0	0	0	0	5	10	5	0	5	NR	NR	0
Rosen 2006	103 ± 23.8	NR	11	2	9	0	0	0	11	0	20	11	9	0	0	0	0
Valvekens 2015	NR	NR	NR	NR	NR	NR	1	0	3	0	NR	NR	NR	NR	NR	NR	NR
Verhagen 2018	NR	NR	NR	NR	NR	NR	27	0	0	0	35	24	3	8	8	0	0
Vuilleumier 2009	58 ± 12.5	1.1 ± 0.3	43	0	43	0	43	0	0	0	86	43	43	0	0	0	0

^a^ NR = Not Reported

### Outcomes of neurectomy surgery

**Fig. 1:**
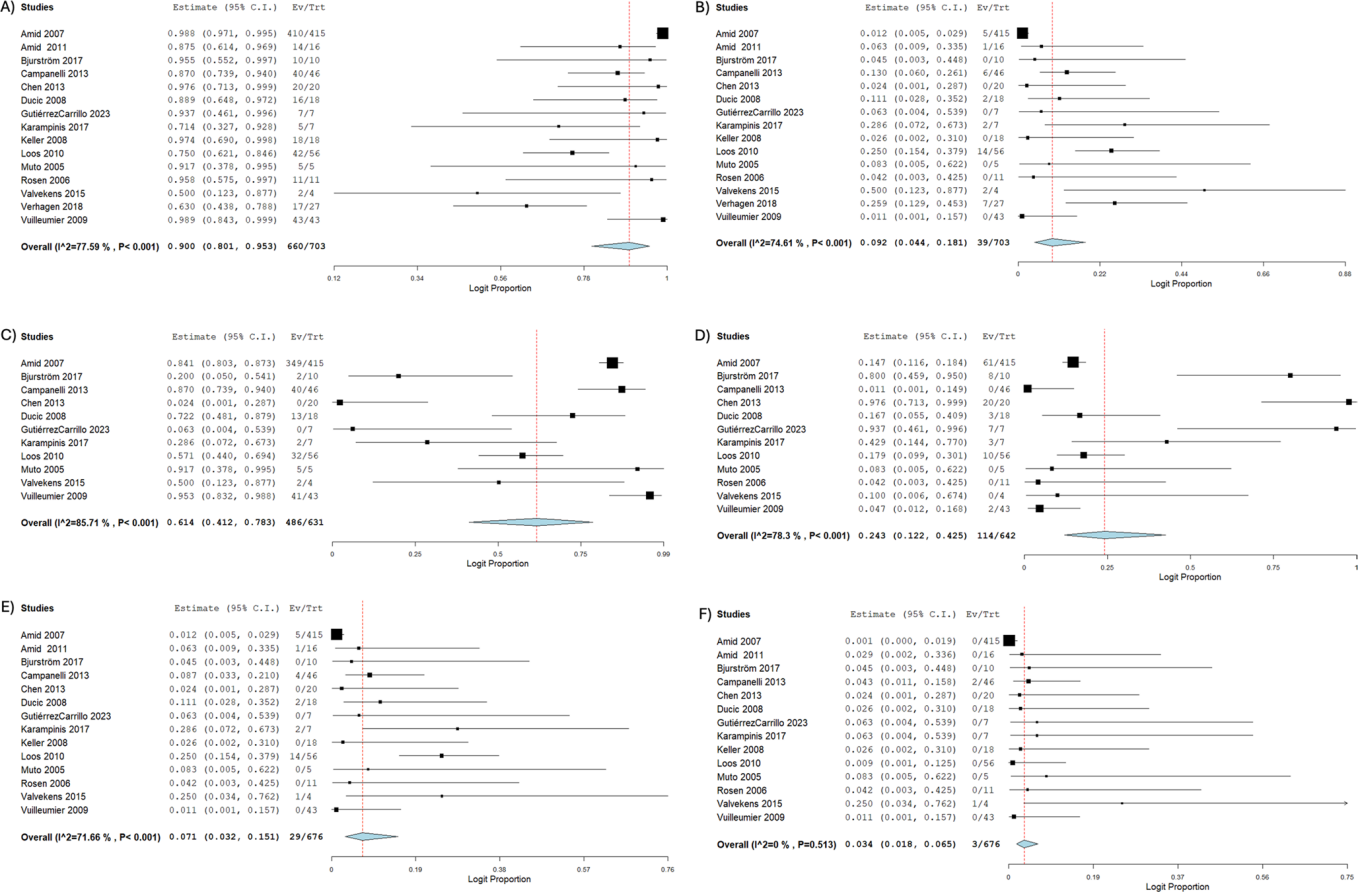
Forrest Plots of pain improvement post-operatively. **A** Overall pain improvement. **B** Overall no pain improvement. **C** Range of improvement (free of pain). **D** Range of improvement (partial improvement). **E** Range of improvement (same level of pain). **F** Range of improvement (pain worsened)

**Fig. 2:**
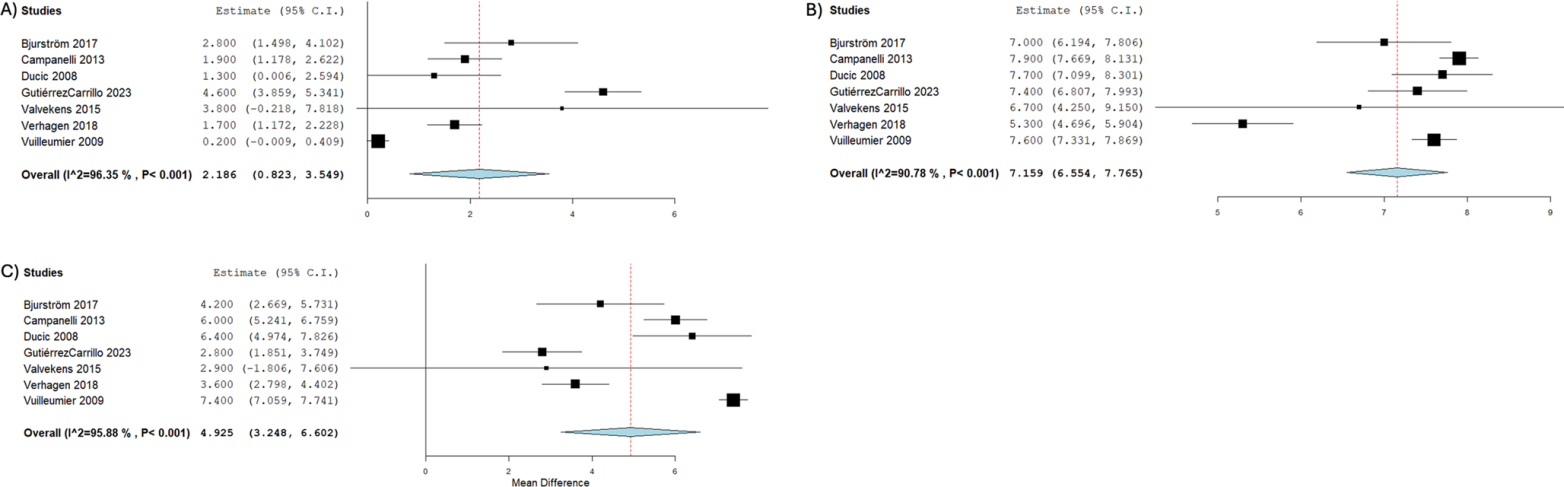
Forrest Plots of VAS scores pre- and post- operatively. **A** Mean VAS Score Pre-neurectomy. **B** Mean VAS Score post-neurectomy. **C** VAS Score mean difference

The outcomes of the neurectomy surgeries are outlined in Table 3. The pooled mean follow-up time was 10.6 months (*n* = 629; 95% CI: 8.39, 12.8; I^2^ = 100%) [1–5, 10–14, 16, 23, 24], with a pooled proportion of patients lost to follow-up at 9.30% (*n* = 683; 95% CI: 7.30, 11.8; I^2^ = 0.00%) [1–5, 10–14, 16, 23–25]. The pooled proportion of inguinal pain improvement post-neurectomy was 90.0% (*n* = 703; 95% CI: 80.1, 95.3; I^2^ = 77.6%) [1–5, 10–14, 16, 22–25], with a pooled proportion of 9.20% not improved post-neurectomy (*n* = 703; 95% CI: 4.40, 18.1; I^2^ = 74.6%) [1–5, 10–14, 16, 22–25]. The overall pain improvement was categorized into four levels: no pain, partial improvement, no change in pain, and worsened pain. The pooled proportion of patients who were free of pain after neurectomy was 61.4% (*n* = 631; 95% CI: 41.2, 78.3; I² = 85.7%) [1–4, 11–14, 16, 23, 25]. Additionally, 24.3% of patients experienced partial improvement (*n* = 642; 95% CI: 12.2, 42.5; I² = 78.3%) [1–4, 11–14, 16, 23–25]. In contrast, 7.10% of patients reported the same level of pain as preoperatively (*n* = 676; 95% CI: 3.2, 15.1; I² = 71.7%) [1–4, 10–14, 16, 22–25], and 3.4% had worse pain post-neurectomy (*n* = 676; 95% CI: 1.18, 6.50; I² = 0.0%) [1–4, 10–14, 16, 22–25]. The Forrest Plots of pain improvement post-operatively are outlined in Fig. 1. In studies that reported pain scales using VAS, the pooled mean VAS score measured pre-neurectomy in 158 groins was 7.16 (95% CI: 6.55, 7.77; I^2^ = 90.8%) [1, 2, 4, 5, 11, 13, 16], and VAS score post-neurectomy after follow-up was 2.19 (*n* = 158; 95% CI: 1.23, 3.15; I^2^ = 96.4%) [1, 2, 4, 5, 11, 13, 16]. The pooled mean difference of VAS shows improved pain scores postoperatively (4.93; *n* = 155; 95% CI: 3.25, 6.60; I^2^ = 95.9%) [1, 2, 4, 5, 11, 13, 16]. The Forrest Plots of VAS score pre- and post- operatively are outlined in Fig. 2.

**Table 3:** Outcome of neurectomy surgeries

Study	No. Patients with Postoperative Complications	Follow-Up		VAS ^b^		Improvement of Pain		Range of Improvement of Pain			
		Lost to Follow-Up	Mean Follow-Up (Months) ± SD	Mean VAS out of 10 Pre-Operation ± SD	Mean VAS out of 10 Post-Operation ± SD	Pain Improvement	Pain Not Improved	Free of Pain	Partial Improvement	Pain the Same	Pain Worsen
Amid 2007	NR ^a^	41	1.4 ± 0	NR	NR	410	5	349	61	5	0
Amid 2011	1	2	1.4 ± 0	NR	NR	14	1	NR	NR	1	0
Bjurström 2017	0	0	6 ± 0	7 ± 1.3	2.8 ± 2.1	10	0	2	8	0	0
Campanelli 2013	NR	0	12 ± 13.5	7.9 ± 0.8	1.9 ± 2.5	40	6	40	0	4	2
Chen 2013	3	0	5.2 ± 1.4	NR	NR	20	0	0	20	0	0
Ducic 2008	0	0	21.3 ± 5.1	7.7 ± 1.3	1.3 ± 2.8	16	2	13	3	2	0
GutiérrezCarrillo 2023	0	0	12 ± 0	7.4 ± 0.8	4.6 ± 1	7	0	0	7	0	0
Karampinis 2017	0	0	14.3 ± 1	NR	NR	5	2	2	3	2	0
Keller 2008	0	NR	NR	NR	NR	18	0	NR	NR	0	0
Loos 2010	4	5	NR	NR	NR	42	14	32	10	14	0
Muto 2005	5	0	31 ± 0	NR	NR	5	0	5	0	0	0
Rosen 2006	0	0	1.5 ± 0	NR	NR	11	0	NR	0	0	0
Valvekens 2015	NR	0	15.6 ± 3.9	6.7 ± 2.5	3.8 ± 4.1	2	2	2	0	1	1
Verhagen 2018	7	3	6 ± 0	5.3 ± 1.6	1.7 ± 1.4	17	7	NR	NR	NR	NR
Vuilleumier 2009	1	4	12 ± 0	7.6 ± 0.9	0.2 ± 0.7	43	0	41	2	0	0

^a^ NR = Not Reported

^b^ VAS = Visual Analog Scale

**Fig. 3:**
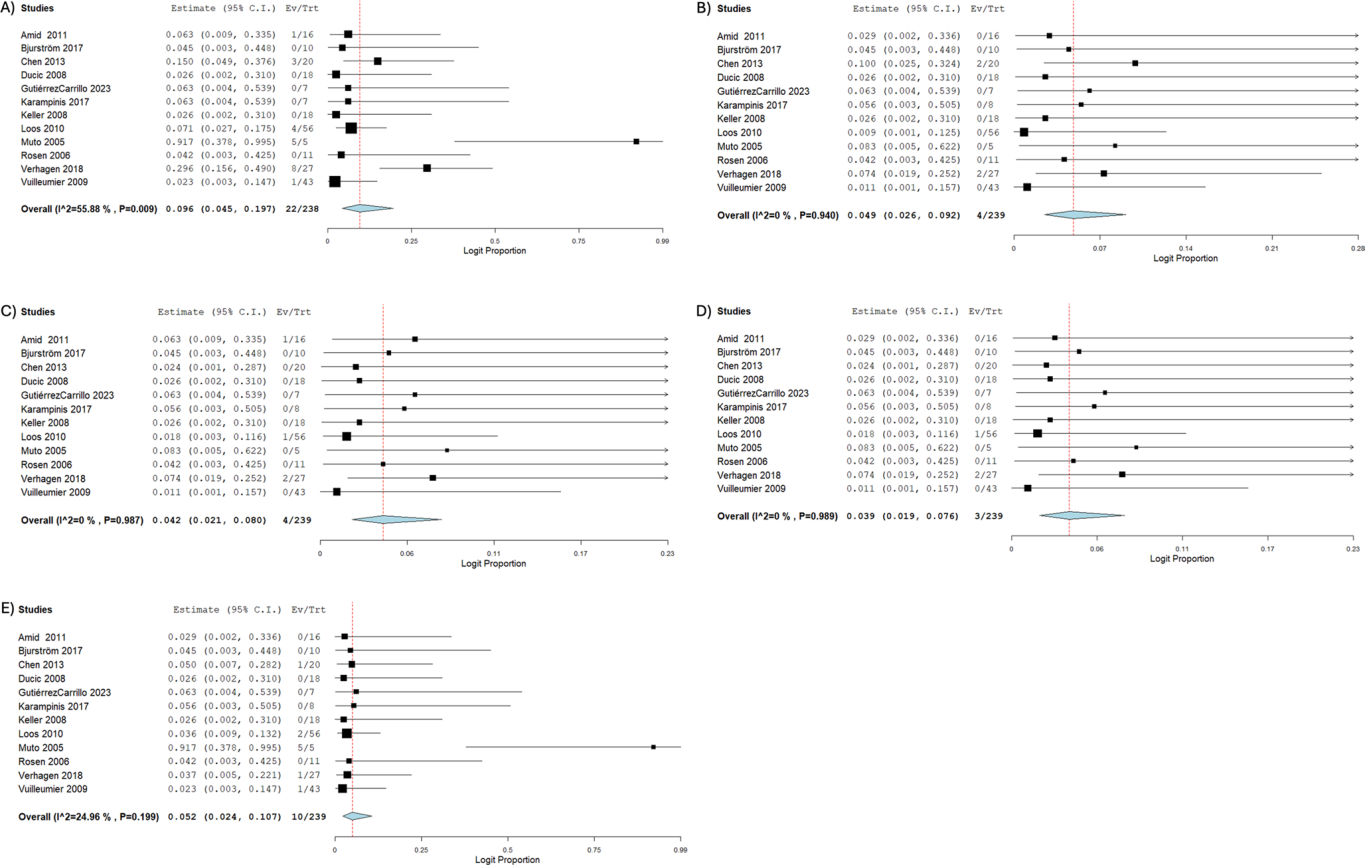
Forrest Plots of total and most common complications. **A** Total Complications. **B** Skin Hyperesthesia. **C** Wound Infection. **D** Seroma and Hematoma. **E** Other

The most observed post-neurectomy complications are outlined in Table 4. The pooled proportion of patients experiencing postoperative complications was 9.40% (*n* = 239; 95% CI: 4.50, 18.7; I^2^ = 51.2%) [1, 3, 5, 10–14, 16, 22, 24, 25]. The top three most common complications were wound infection, seroma/hematoma, and skin hyperesthesia. Specifically, the pooled proportion of patients with wound infection post-neurectomy was 4.20% (*n* = 239; 95% CI: 2.10, 8.00; I^2^ = 0.00%) [1, 3, 5, 10–14, 16, 22, 24, 25], seroma/hematoma was 3.90% (*n* = 239; 95% CI: 1.90, 7.60; I^2^ = 0.00%) [1, 3, 5, 10–14, 16, 22, 24, 25], and skin hyperesthesia was 4.90% (*n* = 239; 95% CI: 2.60, 9.20; I^2^ = 0.00%) [1, 3, 5, 10–14, 16, 22, 24, 25]. Other types of complications were observed in 5.20% of patients (*n* = 239; 95% CI: 2.40, 10.7; I^2^ = 24.9%) [1, 3, 5, 10–14, 16, 22, 24, 25]. The Forrest Plots of total and most common complications are outlined in Fig. 3.

**Table 4:** Most observed Post-Neurectomy complications

Study	No. Patients with Postoperative Complications	Wound Infection	Seroma/Hematoma	Skin Hyperesthesia	Others ^b^
Amid 2007	NR ^a^	NR	NR	NR	NR
Amid 2011	1	1	0	0	0
Bjurström 2017	0	0	0	0	0
Campanelli 2013	NR	NR	NR	NR	NR
Chen 2013	3	0	0	2	1
Ducic 2008	0	0	0	0	0
GutiérrezCarrillo 2023	0	0	0	0	0
Karampinis 2017	0	0	0	0	0
Keller 2008	0	0	0	0	0
Loos 2010	4	1	1	0	2
Muto 2005	5	0	0	0	5
Rosen 2006	0	0	0	0	0
Valvekens 2015	NR	NR	NR	NR	NR
Verhagen 2018	7	2	2	2	1
Vuilleumier 2009	1	0	0	0	1

^a^ NR = Not Reported

^b^ Others = Significant Blood Loss + Transfusion + Embolization + Intraoperative Laceration + Loss of Cremasteric Reflex + Post-Op Recurrent Hernia + Diaphragm Lesion + Ischemic Orchitis/Orchidectomy

### Subgroup analysis of neurectomy surgery

We conducted a subgroup analysis as part of our meta-analysis to investigate potential differences in the effects of neurectomy surgery across various groups. The included subgroups are the types of neurectomy (triple and double) and the approaches to neurectomy (open, endoscopic retroperitoneal, combined), reflecting on the improvement in patients’ chronic pain.

The outcomes of subgroup analysis are outlined in Table 5. For the double neurectomy type, the pooled mean VAS score measured pre-neurectomy in 49 groins was 7.96 (95% CI: 7.09, 8.83; I² = 75.4%) [11, 13]. The VAS score post-neurectomy after follow-up was 0.88 (*n* = 49; 95% CI: −1.36, 3.12; I² = 52.1%) [11, 13]. The pooled mean difference of VAS score was 7.39 (*n* = 49; 95% CI: 7.04, 7.72; I² = 00.0%) [11, 13]. The pooled proportion of inguinal pain improvement post-neurectomy was 76.9% (*n* = 61; 95% CI: 38.5, 94.6; I^2^ = 60.8%) [10–14], with a pooled proportion of 23.1% not improved post-neurectomy (*n* = 61; 95% CI: 5.40, 61.5; I^2^ = 60.8%) [10–14]. The overall pain improvement ranged from free of pain, partial improvement, pain the same, and pain worse. The pooled proportion of patients free of pain post-neurectomy was 80.1% (*n* = 59; 95% CI: 42.5, 95.6; I^2^ = 70.0%) [11–14], and 7.40% had partial improvement (*n* = 59; 95% CI: 2.80, 18.3; I^2^ = 00.0%) [11–14]. Meanwhile, 20.1% of patients experienced the same level of pain as preoperative (*n* = 61; 95% CI: 5.70, 51.1; I^2^ = 48.4%) [10–14], and 8.60% had worse pain post-neurectomy (*n* = 61; 95% CI: 2.20, 28.2; I^2^ = 19.96%) [10–14]. The pooled proportion of patients experiencing postoperative complications was 15.3% (*n* = 60; 95% CI: 1.30, 71.6; I^2^ = 75.19%) [11–14]. For the triple neurectomy type, the pooled mean VAS score measured pre-neurectomy in 25 groins was 7.33 (95% CI: 6.91, 7.76; I^2^ = 00.0%) [1, 13, 16], and VAS score post-neurectomy after follow-up was 2.73 (*n* = 25; 95% CI: 24.4, 5.21; I^2^ = 94.9%) [1, 13, 16]. The pooled mean difference of VAS score was 4.59 (*n* = 25; 95% CI: 2.09, 7.08; I^2^ = 91.6%) [1, 13, 16]. The pooled proportion of inguinal pain improvement post-neurectomy was 98.2% (*n* = 474; 95% CI: 96.3, 99.1; I^2^ = 00.0%) [1, 3, 10, 13, 16, 23], with a pooled proportion of 1.8% not improved post-neurectomy (*n* = 474; 95% CI: 0.90, 3.70; I^2^ = 00.0%) [1, 3, 10, 13, 16, 23]. The overall pain improvement ranged from free of pain, partial improvement, pain the same, and pain worse. The pooled proportion of patients free of pain post-neurectomy was 30.6% (*n* = 460; 95% CI: 5.90, 75.7; I^2^ = 89.5%) [1, 3, 13, 16, 23], and 69.2% had partial improvement (*n* = 460; 95% CI: 23.2, 94.4; I^2^ = 90.0%) [1, 3, 13, 16, 23]. Meanwhile, 1.80% of patients experienced the same level of pain as preoperative (*n* = 474; 95% CI: 0.90, 3.70; I^2^ = 00.0%) [1, 3, 10, 13, 16, 23], and 2.30% had worse pain post-neurectomy (*n* = 474; 95% CI: 0.70, 7.40; I^2^ = 11.15%) [1, 3, 10, 13, 16, 23]. The pooled proportion of patients experiencing postoperative complications was 10.5% (*n* = 45; 95% CI: 4.20, 24.0; I^2^ = 00.0%) [1, 3, 13, 16].

**Table 5:** Outcomes of subgroup analysis

Subgroup	Sample size *N*	Pooled Estimate (95% CI) ^a^	I² (%) ^b^
Pre-Neurectomy VAS Score ^c^			
Double Neurectomy	49	7.96 (7.09, 8.83)	75.4
Triple Neurectomy	25	7.33 (6.91, 7.76)	0
Open Neurectomy	134	7.16 (6.33, 7.99)	95.2
Combined Approach	N/A	N/A	N/A
End. Retro. Approach ^d^	N/A	N/A	N/A
Pain Improvement			
Double Neurectomy	61	76.9% (38.5, 94.6)	60.8
Triple Neurectomy	474	98.2% (96.3, 99.1)	0
Open Neurectomy	621	89.9% (74.2, 96.5)	88.6
Combined Approach	32	87.3% (29.5, 99.1)	69.6
End. Retro. Approach	35	95.50% (80.6, 99.1)	0
Partial Improvement			
Double Neurectomy	59	7.40% (2.80, 18.3)	0
Triple Neurectomy	460	69.2% (23.2, 94.4)	90
Open Neurectomy	578	12.9% (8.00, 20.2)	45.3
Combined Approach	14	7.10% (1.00, 37.3)	0
End. Retro. Approach	35	72.2% (12.2, 98.0)	77.9
Pain worse			
Double Neurectomy	61	8.60% (2.20, 28.2)	19.96
Triple Neurectomy	474	2.30% (0.70, 7.40)	11.15
Open Neurectomy	594	1.70% (0.60, 4.70)	14.4
Combined Approach	32	8.70% (1.50, 37.9)	30.84
End. Retro. Approach	35	4.50% (0.90, 19.4)	0
Subgroup	Sample size *N*	Pooled Estimate (95%CI)	I²(%)
Post-Neurectomy VAS Score			
Double Neurectomy	49	0.88 (−1.36, 3.12)	52.1
Triple Neurectomy	25	2.73 (24.4, 5.21)	94.9
Open Neurectomy	134	1.25 (0.19, 2.31)	93.1
Combined Approach	N/A	N/A	N/A
End. Retro. Approach	N/A	N/A	N/A
Pain Free			
Double Neurectomy	59	80.1% (42.5, 95.6)	70
Triple Neurectomy	460	30.6% (5.90, 75.7)	89.5
Open Neurectomy	578	81.1% (66.2, 90.4)	85.6
Combined Approach	N/A	N/A	N/A
End. Retro. Approach	35	27.8% (2.00, 87.8)	77.9
Same Pain			
Double Neurectomy	61	20.1% (5.70, 51.1)	48.4
Triple Neurectomy	474	1.80% (0.90, 3.70)	0
Open Neurectomy	594	6.10% (1.70,19.9)	87.4
Combined Approach	32	8.7% (1.50, 37.9)	30.8
End. Retro. Approach	35	4.50% (0.90, 19.4)	0
Complications			
Double Neurectomy	60	15.3% (1.30, 71.6)	75.19
Triple Neurectomy	45	10.5% (4.20, 24.0)	0
Open Neurectomy	160	8.00% (2.80, 20.5)	62.1
Combined Approach	29	3.30% (0.50, 20.1)	0
End. Retro. Approach	35	28.7% (2.70, 85.3)	75.6

^a^ CI = Confidence Interval

^b^ I² = Heterogeneity

^c^ VAS = Visual Analog Scale

^d^ End. Retro. Approach = Endoscopic Retroperitoneal Approach

For the open approach neurectomy, the pooled mean VAS score measured pre-neurectomy in 134 groins was 7.16 (95% CI: 6.33, 7.99; I^2^ = 95.19%) [2, 5, 11, 13], and VAS score post-neurectomy after follow-up was 1.25 (*n* = 134; 95% CI: 0.19, 2.31; I^2^ = 93.1%) [2, 5, 11, 13]. The pooled mean difference of the VAS score was 5.85 (*n* = 134; 95% CI: 4.03, 7.67; I^2^ = 96.1%) [2, 5, 11, 13]. The pooled proportion of inguinal pain improvement post-neurectomy was 89.9% (*n* = 621; 95% CI: 74.2, 96.5; I^2^ = 88.6%) [2, 5, 10, 11, 13, 23, 25], with a pooled proportion of 8.60% not improved post-neurectomy (*n* = 621; 95% CI: 3.00, 22.2; I^2^ = 86.8%) [2, 5, 10, 11, 13, 23, 25]. The pooled proportion of patients free of pain post-neurectomy was 81.1% (*n* = 578; 95% CI: 66.2, 90.4; I^2^ = 85.6%) [2, 11, 13, 23, 25], and 12.9% had partial improvement (*n* = 578; 95% CI: 8.00, 20.2; I^2^ = 45.3%) [2, 11, 13, 23, 25]. Meanwhile, 6.10% of patients experienced the same level of pain as preoperative (*n* = 594; 95% CI: 1.70, 19.9; I^2^ = 87.4%) [2, 10, 11, 13, 23, 25], and 1.70% had worse pain post-neurectomy (*n* = 594; 95% CI: 0.60, 4.70; I^2^ = 14.4%) [2, 10, 11, 13, 23, 25]. The pooled proportion of patients experiencing postoperative complications was 8.00% (*n* = 160; 95% CI: 2.80, 20.5; I^2^ = 62.1%) [5, 10, 11, 13, 25]. For the combined neurectomy approach, the pooled proportion of inguinal pain improvement post-neurectomy was 87.3% (*n* = 32; 95% CI: 29.5, 99.1; I^2^ = 69.6%) [4, 22, 24]. With a pooled proportion of 12.7% not improved post-neurectomy (*n* = 32; 95% CI: 0.90, 70.5; I^2^ = 69.6%) [4, 22, 24]. However, the pooled proportion of patients with partial improvement was 7.1% (*n* = 14; 95% CI: 1.00, 37.3; I^2^ = 00.0%) [4, 24]. Meanwhile, 8.7% of patients experienced the same level of pain as preoperative (*n* = 32; 95% CI: 1.50, 37.9; I^2^ = 30.8%) [4, 22, 24], and 8.70% had worse pain post-neurectomy (*n* = 32; 95% CI: 1.50, 37.9; I^2^ = 30.84%) [4, 22, 24]. The pooled proportion of patients experiencing postoperative complications was 3.30% (*n* = 29; 95% CI: 0.50, 20.1; I^2^ = 00.0%) [22, 24]. For the endoscopic retroperitoneal neurectomy approach, the pooled proportion of inguinal pain improvement post-neurectomy was 95.50% (*n* = 35; 95% CI: 80.6, 99.1; I^2^ = 00.0%) [1, 3, 12], with a pooled proportion of 4.50% not improved post-neurectomy (*n* = 35; 95% CI: 0.90, 19.4; I^2^ = 00.0%) [1, 3, 12]. The pooled proportion of patients free of pain post-neurectomy was 27.8% (*n* = 35; 95% CI: 2.00, 87.8; I^2^ = 77.9%) [1, 3, 12], and 72.2% had partial improvement (*n* = 35; 95% CI: 12.2, 98.0; I^2^ = 77.9%) [1, 3, 12]. Meanwhile, 4.50% of patients experienced the same level of pain as preoperative (*n* = 35; 95% CI: 0.90, 19.4; I^2^ = 00.0%) [1, 3, 12], and 4.50% had worse pain post-neurectomy (*n* = 35; 95% CI: 0.90, 19.4; I^2^ = 00.0) [1, 3, 12]. The pooled proportion of patients experiencing postoperative complications was 28.7% (*n* = 35; 95% CI: 2.70, 85.3; I^2^ = 75.6%) [1, 3, 12].

## Discussion

The search for the optimal surgical treatment for chronic pain after inguinal hernia repair surgery is ongoing. To our knowledge, this is the most comprehensive systematic and meta-analysis review that focuses on assessing the effectiveness of neurectomy on various surgical approaches: open neurectomy, laparoscopic transabdominal endoscopic retroperitoneal neurectomy, combined neurectomy, and the types of neurectomy—single, double and triple. Overall, the vast majority of the patients experienced pain improvement post-neurectomy, with a significant portion became entirely pain-free. Only a small minority saw no benefit.

We conducted a subgroup analysis to assess the effects of each type of neurectomy. However, due to limited data, only double and triple neurectomy were included. Both types appear effective in reducing inguinal pain; however triple neurectomy showed superior results, with a higher overall improvement rate (98.2% vs. 76.9%) and a lower percentage of patients reporting worsened pain levels postoperatively (2.3% vs. 8.6%) compared to double neurectomy. Interestingly, 80.1% of the patients who had double neurectomy were completely free of pain. The literature shows that the ilioinguinal nerve and the iliohypogastric nerve are the most affected and are usually the most resected, as confirmed by our results [11]. Furthermore, it is suggested, in accordance with other studies, that symptoms inconsistent with injury to the genital branch of the genitofemoral nerve, coupled with no intraoperative findings indicating such injury, do not warrant resection, especially since neurectomy of this branch can cause injury to the spermatic blood vessels [11, 27–30]. In contrast, specialist centers recommend a standard “triple neurectomy” for this pain syndrome, as consistently identifying the affected nerve is extremely difficult, if not impossible [1, 3, 10, 31]. This is due to central and peripheral neural communication and the frequent involvement of multiple nerves during hernia surgery. Furthermore, in one stage anterior triple neurectomy, mobilization of the spermatic cord can be avoided [1, 3, 10, 31]. A more rigorously designed randomized controlled trial (RCT) is needed to provide accurate evidence on this dilemma. Until then, the triple neurectomy remains the standard treatment for neurectomy in CPIP.

The analysis of the surgical approaches, which included open, combined, laparoscopic transabdominal, and endoscopic retroperitoneal approaches, disclosed that all were effective for CPIP but with distinct profile. The open approach showed balanced efficacy: 89.9% improvement and 8% complications. However, this approach had several challenges, including reoperation in a previously scarred area, difficulty in locating the three target nerves, increasing the likelihood of injury to the spermatic cord and testicle, and disrupting an already sensitive area [16, 30]. Though this approach has a high rate of complete pain relief post-neurectomy at 81.1%. On the other hand, the combined approach provided high diagnostic accuracy to concordant pathologies such as recurrent hernia and misplaced mesh [4, 22], and had the lowest complication rate among approaches at 3.3%. However, its overall improvement was 87.3%, which, while like the ranges reported in previously published studies (60–87%) [4, 32], was the lowest among our subgroups. This suggests that it can be safe but less effective. The small sample size and wide confidence interval of this approach warrant cautious interpretation of the results. The endoscopic retroperitoneal approach demonstrated the highest overall improvement, 95.5%, among our subgroups. However, only 27.8% of patients reported being completely pain-free. Despite excellent nerve visualization [33], the procedure remains challenging, even for experts, as anatomical variances can complicate nerve identification [33]. Additionally, it carries a notable complication rate of 28.7%. This may reflect one study’s findings where cremasteric reflex loss (classified by authors as a side effect) occurred in all patients [12]. However, in our analysis, we classified this as a complication due to its potential implications for recovery and quality of life. Standardized complication criteria and larger comparative studies are needed to optimize approach selection, particularly for minimally invasive techniques where current data remains limited.

It is important to note that neurectomy was not the only factor contributing to pain relief, as the impact of other factors, such as the removal of mesh (meshectomy), cannot be ruled out due to limited data. Research on meshectomy’s effect on chronic post-inguinal pain (CPIP) has shown inconsistent results. Some studies suggest that the implementation of mesh itself—regardless of nerve entrapment—can cause inguinal pain [34–36]. Other old studies suggest that the use of mesh does not contribute to postherniorrhaphy chronic pain [15, 37–40]. A recent study highlights that inguinodynia can have heterogeneous causes, where neuropathic pain may be treated effectively with neurectomy, but nociceptive pain often requires meshectomy [41]. These findings underscore the importance of developing tailored treatment approaches based on the specific source of pain. The inconsistency of these findings highlights the need for further research, particularly with standardized methodologies and better control for confounding factors.

While many patients report improvements in inguinal pain following neurectomy, it is crucial to consider the specific complications and variability in outcomes. Our analysis indicated that 9.40% of patients experienced postoperative complications. The most common issues included skin hyperesthesia (4.9%), wound infection (4.2%), and seroma/hematoma (3.9%). Other complications, such as significant blood loss, transfusions, embolization, intraoperative laceration, loss of the cremasteric reflex, postoperative recurrent hernia, diaphragm lesions, and ischemic orchitis requiring orchidectomy, collectively accounted for 5.20%. Despite the relatively low incidence of these complications, they can contribute to the overall risk of residual pain. In our study, 7.10% of patients continued to experience severe pain similar to preoperative levels, despite adequate neurectomy. Additionally, 3.4% reported that their pain worsened post-neurectomy. Changes in sensation, particularly skin hyperesthesia or hypersensitivity following neurectomy, are reported as the most common complication, with a pooled estimate of 4.9%. While some patients showed resolution within four weeks, others required up to six months to improve, with some patients reporting persistent hypersensitivity lasting over a year, albeit with reduced intensity [42]. Gangopadhyay N studied peripheral nerve injury and neuropathic pain in a rat model, suggesting that hyperalgesia arises from central neuron sensitization, leading to an enhanced response to normal stimuli, as well as peripheral sensitization at sensory nerve endings due to interactions between injured and normal nerve fibers [43]. Contributing factors may include surgical technique and individual variables, such as preoperative pain levels and central sensitization [3, 5, 42]. The risk of pain due to deafferentation may be diminished when terminal, rather than proximal neurectomies, are preferred, as the probability of accidentally severing afferent axons diminishes distally [2].

Several limitations must be considered. One key limitation is the lack of a gold-standard diagnostic tool for neuropathic pain across the studies included, which could introduce measurement bias. Additionally, the high levels of heterogeneity among the included studies present significant challenges for meaningful interpretation. This is especially true given the variability in the definitions of chronic pain duration, differences in baseline characteristics, and the range of procedural methods used across patient populations. The studies also employed various combinations of neurectomy types and approaches, leading to persistent heterogeneity even after subgroup analyses. Moreover, the diverse assessment tools used to evaluate pain contributed to inconsistent outcomes. Nine of the studies included were identified as having a high risk of bias, and several retrospective designs raised concerns about recall bias, further complicating our findings. Finally, the inconsistent definitions of “improvement” across studies limit the reliability of our conclusions.

## Conclusion

This meta-analysis represents the most comprehensive evaluation of neurectomy as treatment for CPIP, showing that most patients experienced pain relief, with many achieving complete resolution. Triple neurectomy was superior to double neurectomy in the overall improvement and lower complication rates. All surgical approaches (open, laparoscopic transabdominal, endoscopic retroperitoneal and combined) were effective, each with distinct profile. Well-designed randomized controlled trials would be of vital importance to facilitate the evolution of surgical strategies and improve the ultimate patient outcome.

## Supplementary Information

Below is the link to the electronic supplementary material.

ESM 1 (PDF 736 KB)

ESM 2 (PDF 1.03 MB)

## Data Availability

With the publication, the data set used for this meta-analysis will be shared upon request from the study authors.

## Abbreviations

CI: Confidence Interval
CPIP: Chronic Postherniorrhaphy Pain
GFN: Genitofemoral Nerve
LFCN: Lateral Femoral Cutaneous Nerve
I²: I-squared (a measure of heterogeneity)
IIN: ilioinguinal nerve
IHN: iliohypogastric nerve
N/A: Not Applicable
PROSPERO: International Prospective Register of Systematic Reviews
PRISMA: Preferred Reporting Items for Systematic Reviews and Meta-Analyses
ROB2: Risk of Bias 2 (for randomized studies)
ROBINS: I-Risk of Bias in Non-randomized Studies-of Interventions
RCT: Randomized Controlled Trial
SD: Standard Deviation
VAS: Visual Analog Scale
