# Supplementary material for: Impact of different neurectomy techniques on managing chronic pain after inguinal hernia repair: a meta-analysis and systematic review
Source: Hernia. 2025 Aug 12;29(1):249. doi: 10.1007/s10029-025-03438-0 (PMC12343651; doi:10.1007/s10029-025-03438-0)

Neurectomy for chronic postoperative pain following hernia repair: An assessment of open, laparoscopic, and endoscopic methods

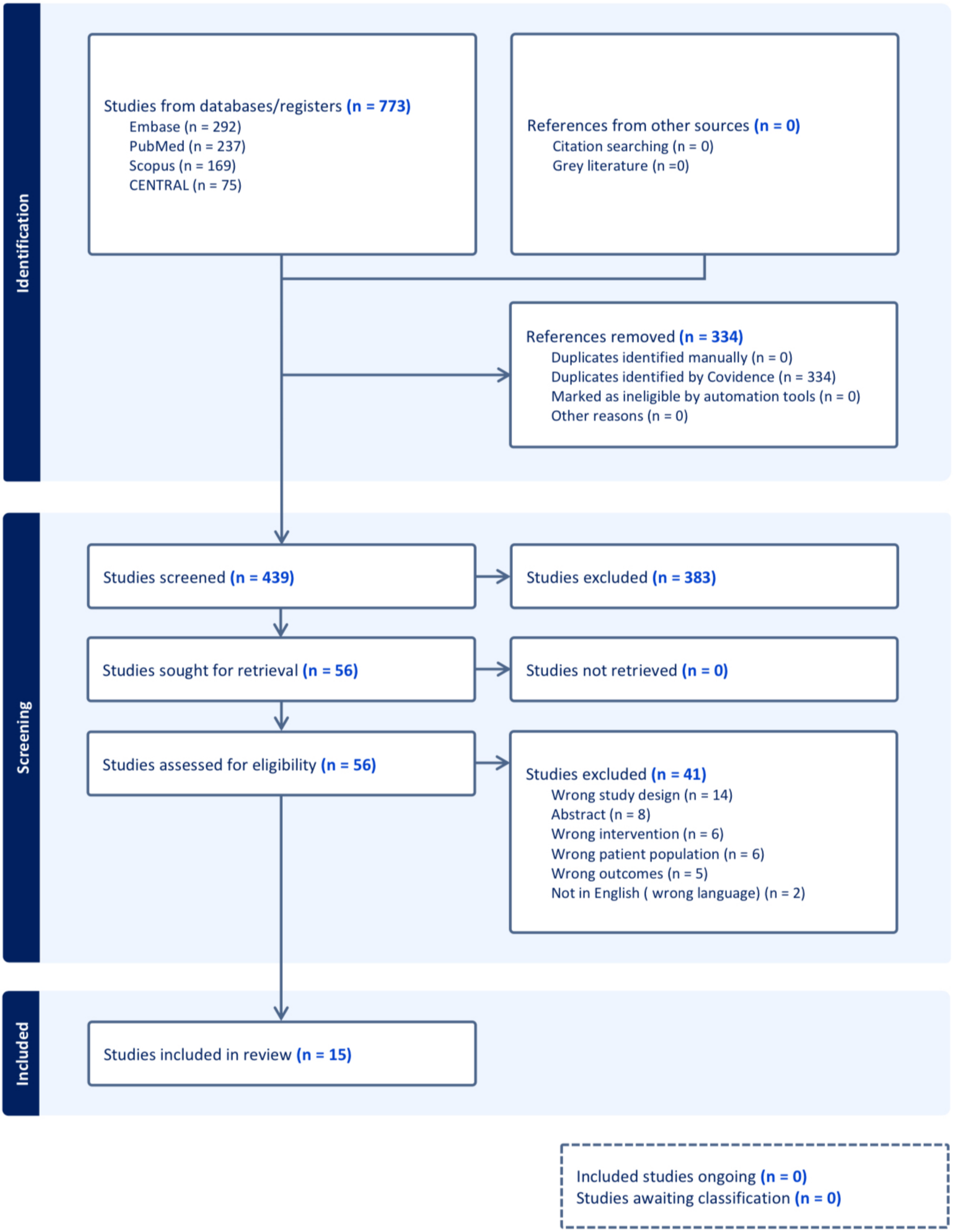

Supplement: Supplementary file 1 — (PDF 736 KB) [file 10029_2025_3438_MOESM1_ESM.pdf]
