# Supplementary material for: Impact of different neurectomy techniques on managing chronic pain after inguinal hernia repair: a meta-analysis and systematic review
Source: Hernia. 2025 Aug 12;29(1):249. doi: 10.1007/s10029-025-03438-0 (PMC12343651; doi:10.1007/s10029-025-03438-0)
Supplement: Supplementary file 2 — (PDF 1.03 MB) [file 10029_2025_3438_MOESM2_ESM.pdf]

A)

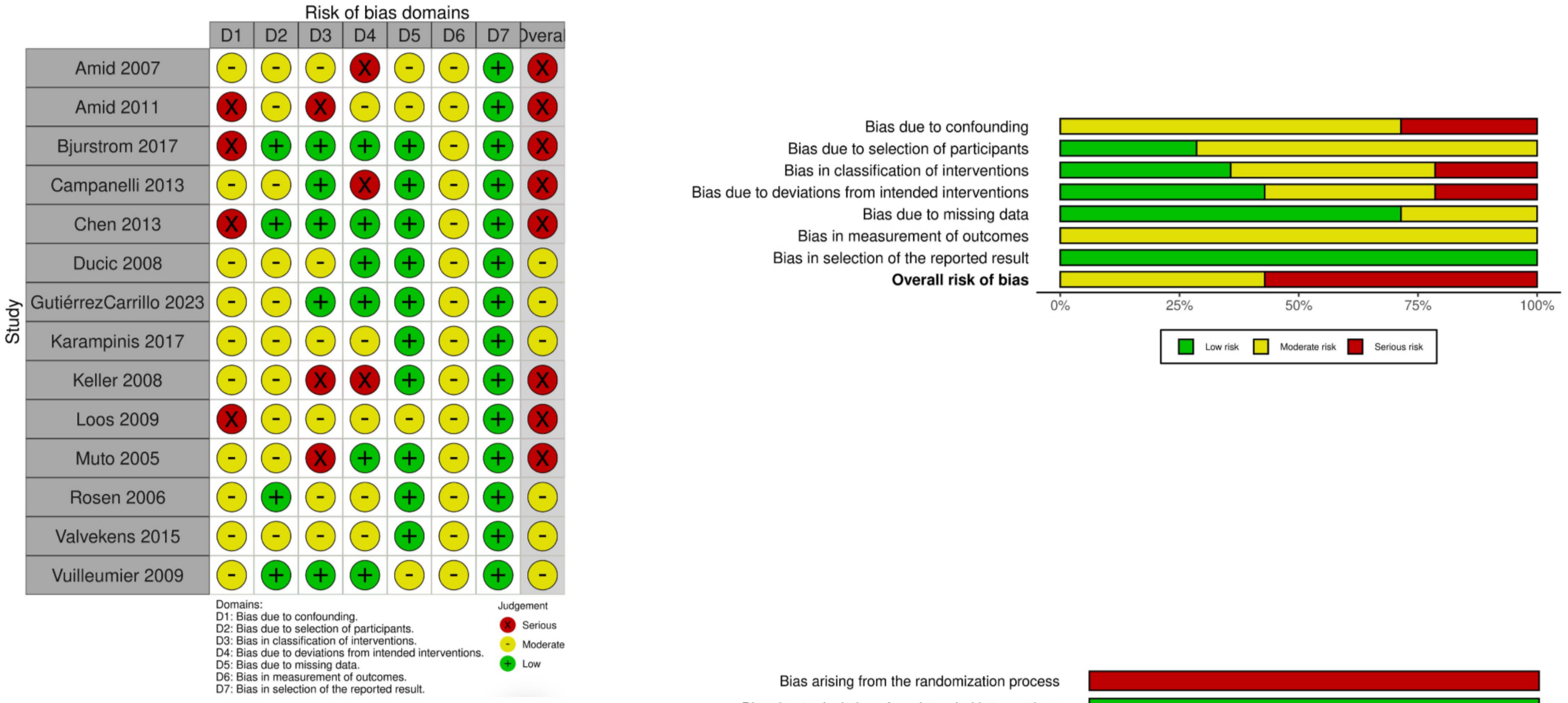

Risk of bias domains
D1
D2
D3
D4
D5
D6
D7
Overall
Study
Amid 2007
Amid 2011
Bjurstrom 2017
Campanelli 2013
Chen 2013
Ducic 2008
GutiérrezCarrillo 2023
Karampinis 2017
Keller 2008
Loos 2009
Muto 2005
Rosen 2006
Valvekens 2015
Vuilleumier 2009
Domains:
D1: Bias due to confounding.
D2: Bias due to selection of participants.
D3: Bias in classification of interventions.
D4: Bias due to deviations from intended interventions.
D5: Bias due to missing data.
D6: Bias in measurement of outcomes.
D7: Bias in selection of the reported result.
Judgement
Serious
Moderate
Low
Bias due to confounding
Bias due to selection of participants
Bias in classification of interventions
Bias due to deviations from intended interventions
Bias due to missing data
Bias in measurement of outcomes
Bias in selection of the reported result
Overall risk of bias
0%
25%
50%
75%
100%
Low risk
Moderate risk
Serious risk

B)

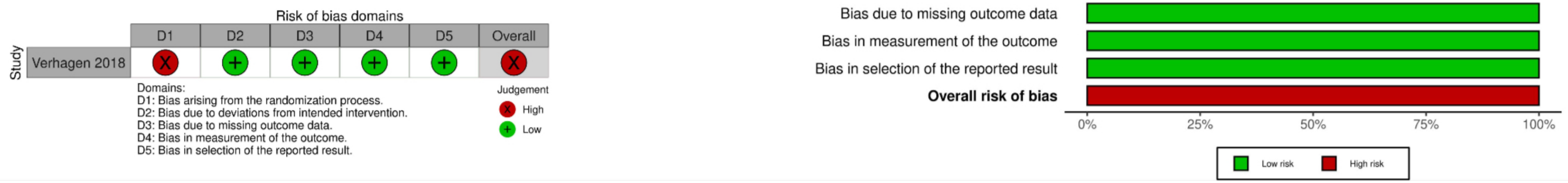

Risk of bias domains
D1
D2
D3
D4
D5
Overall
Study
Verhagen 2018
Domains:
D1: Bias arising from the randomization process.
D2: Bias due to deviations from intended intervention.
D3: Bias due to missing outcome data.
D4: Bias in measurement of the outcome.
D5: Bias in selection of the reported result.
Judgement
High
Low
Bias arising from the randomization process
Bias due to deviations from intended interventions
Bias due to missing outcome data
Bias in measurement of the outcome
Bias in selection of the reported result
Overall risk of bias
0%
25%
50%
75%
100%
Low risk
High risk
